# Identification of potential biomarkers for ankylosing spondylitis based on bioinformatics analysis

**DOI:** 10.1186/s12891-023-06550-3

**Published:** 2023-05-24

**Authors:** Dongxu Li, Ruichao Cao, Wei Dong, Minghuang Cheng, Xiaohan Pan, Zhenming Hu, Jie Hao

**Affiliations:** 1 Department of Orthopedics, The First Affiliated Hospital of Chongqing Medical University, Yuzhong, Chongqing, China; 2 Orthopedic Laboratory of Chongqing Medical University, Yuzhong, Chongqing, China

**Keywords:** Ankylosing spondylitis, Immune infiltration, Bioinformatics, Biomarkers, GWAS

## Abstract

**Objective:**

The aim of this study was to search for key genes in ankylosing spondylitis (AS) through comprehensive bioinformatics analysis, thus providing some theoretical support for future diagnosis and treatment of AS and further research.

**Methods:**

Gene expression profiles were collected from Gene Expression Omnibus (GEO, http://www.ncbi.nlm.nih.gov/geo/) by searching for the term "ankylosing spondylitis". Ultimately, two microarray datasets (GSE73754 and GSE11886) were downloaded from the GEO database. A bioinformatic approach was used to screen differentially expressed genes and perform functional enrichment analysis to obtain biological functions and signalling pathways associated with the disease. Weighted correlation network analysis (WGCNA) was used to further obtain key genes. Immune infiltration analysis was performed using the CIBERSORT algorithm to conduct a correlation analysis of key genes with immune cells. The GWAS data of AS were analysed to identify the pathogenic regions of key genes in AS. Finally, potential therapeutic agents for AS were predicted using these key genes.

**Results:**

A total of 7 potential biomarkers were identified: DYSF, BASP1, PYGL, SPI1, C5AR1, ANPEP and SORL1. ROC curves showed good prediction for each gene. T cell, CD4 naïve cell, and neutrophil levels were significantly higher in the disease group than in the paired normal group, and key gene expression was strongly correlated with immune cells. CMap results showed that the expression profiles of ibuprofen, forskolin, bongkrek-acid, and cimaterol showed the most significant negative correlation with the expression profiles of disease perturbations, suggesting that these drugs may play a role in AS treatment.

**Conclusion:**

The potential biomarkers of AS screened in this study are closely related to the level of immune cell infiltration and play an important role in the immune microenvironment. This may provide help in the clinical diagnosis and treatment of AS and provide new ideas for further research.

**Supplementary Information:**

The online version contains supplementary material available at 10.1186/s12891-023-06550-3.

## Introduction

Ankylosing spondylitis (AS) is an immune-related chronic inflammatory disease. In the more severe stage of the disease, inflammation can lead to fibrosis and calcification of spinal joints and loss of flexibility and fusion, causing severe pain and disability and bringing significant physical and social burden to patients [1]. In addition to back pain and progressive spinal ankylosis, extra-articular symptoms include psoriasis, inflammatory bowel illness, and acute anterior uveitis [2]. The prevalence of AS varies by region, from 0.74% in Africa to 3.19% in North America [3]. In addition to ethnic factors, the incidence of AS has shown genetic and gender-related associations [4], and earlier research has highlighted the significance of immunological and genetic variables in AS, specifically, the close relationship between AS and HLA-B27, but the overall genetic risk associated with HLA-B27 in AS is less than one-third, suggesting that there may be other factors influencing the occurrence and development of AS and that the aetiology of AS remains unclear [5, 6]. Because the onset of AS is insidious and it is difficult to detect obvious imaging manifestations in the early stage, the diagnosis is often delayed. It is estimated that patients have presented symptoms for approximately 1 year at the time of referral in the United States, and the delay in referral in Western Europe and the rest of the world may be more than 3 years [7], a situation that usually leads to delays in AS treatment and loss of the optimal time for treatment.

Currently, AS is not completely curable, and pharmacological treatment remains the mainstay treatment for AS, with some patients with severe disease requiring surgical intervention [1, 8]. The traditional treatment is nonsteroidal anti-inflammatory drugs and physical therapy. In recent years, anti-tumour necrosis factor drugs and IL-23/17 pathway inhibitors have been increasingly used in clinical practice [9]. However, the treatment of AS is difficult, and most patients need lifelong medication to control their symptoms. Therefore, the search for new diagnostic and therapeutic targets is urgently needed. By uncovering and elucidating the molecular mechanisms of AS, it is possible to identify new avenues for future AS treatment.

Bioinformatics analysis is increasingly used for microarray data analysis to identify disease markers [9]. Weighted gene coexpression network analysis (WGCNA) is a new systems biology approach for identifying candidate biomarkers or therapeutic targets [10]. To search for potential markers of AS and the specific signalling pathways involved, we performed WGCNA on two AS microarray datasets in the GEO database. Genome-wide association studies (GWAS) have identified genetic variants that affect many complex traits [11]. To identify the pathogenic regions of key genes in ankylosing spondylitis, we used AS GWAS data to identify SNP pathogenic regions corresponding to key genes. The relationship between differentially expressed genes (DEGs) and immune infiltration was analysed using the CIBERSORT algorithm [12]. To further explore the molecular mechanism of core genes, we performed gene set enrichment analysis (GSEA) to investigate the relationship between key genes and pathogenic genes and performed regulatory network analysis of key genes. Finally, we predicted the drugs that may have therapeutic effects on AS through the CMap database. We believe this study will help to identify new potential biomarkers and new therapeutic approaches for AS.

## Materials and methods

### Prepare the data

The GSE73754 data file was downloaded from the NCBI GEO public database; the annotation platform was GPL10558, with a total of 72 sets of transcriptome data containing a control group (n = 20) and disease group (n = 52). The series matrix file data file of GSE11886 was downloaded, and the annotation platform was GPL570, with a total of 17 groups of transcriptome data, including a control group (n = 9) and disease group (n = 8). The SVA algorithm was applied for batch correction of data between the chip, and limma package was used to identify molecular mechanisms associated with disease, with genetic screening conditions of | logFC |> 0.585 and *p* values < 0.05.

### Functional enrichment analyses of DEGs

The Metascape database (www.metascape.org) was used for annotation and visualization to obtain the biological functions and signalling pathways involved in the disease occurrence process. Gene Ontology (GO) analysis and Kyoto Encyclopedia of Genes and Genomes (KEGG) pathway analysis were performed for specific genes [13]. A minimum overlap number ≥ 3 and *p* ≤ 0.05 were considered statistically significant.

### Weighted gene coexpression network analysis (WGCNA)

The WGCNA-R package was used to construct a coexpression network of all genes in the downloaded dataset. The soft threshold was set to 7, and 10 000 genes with the largest variance were screened for the next analysis. WGCNA networks were constructed to explore relevant coexpression networks in AS [10]. An ROC curve was used to verify the diagnostic efficacy, and the AUC value was positively correlated with the prediction effect.

### GWAS analysis

The Gene Atlas database is a large database documenting associations between hundreds of traits and millions of variants with the use of the UK Biobank cohort. These associations were calculated using 452,264 UK individuals in the UK Biobank database, encompassing a total of 778 phenotypes with a total of 30 million loci [11].

### Immunoinfiltration analysis

The CIBERSORT method can be used to identify 22 human immune cell phenotypes, including T cell, B cell, plasma cell and myeloid cell subsets. In this study, the CIBERSORT algorithm was used to analyse the data in the public database, the relative proportion of 22 immune infiltrating cells was obtained using the algorithm, and the correlation between gene expression and immune cell content was analysed [12].

### GSEA

Core genes were ranked according to their differential expression in the two classes of samples using a predefined set of genes, and then, whether the predefined set of genes was enriched at the top or bottom of this ranking table was checked. In this study, GSEA was used to compare differential signalling pathways between the high expression group and the low expression group and to explore the molecular mechanism of core genes in the two groups of patients.

### Analysis of the regulatory network of core genes

All calculations performed using RcisTarget are based on motifs. The standardized enrichment scores (NES) for motifs depend on the total number of motifs in the database. In addition to the motifs annotated using the source data, we also inferred further annotation files based on motif similarity and gene sequence. The overexpression of each motif in a gene set was estimated by calculating the area under the curve (AUC) for each motif-motif pair. The NES of each motif was calculated from the AUC distribution of all motifs in the gene set [14].

### CMap drug prediction

Connectivity Map (CMap) [15] is an intervention gene expression profile database. It is mainly used to reveal functional associations between small molecule compounds, genes, and disease states. It contains 1309 microarray data sets from five human tumour cell lines before and after treatment with small molecule drugs. We used DEGs in AS to predict potential targeted therapeutic agents.

### Statistical analysis

Statistical analyses were performed with R, version 4.0, and *p* values of less than 0.05 were considered to indicate statistical significance.

## Results and discussion

### Screening of differentially expressed genes (DEGs)

**Fig. 1:**
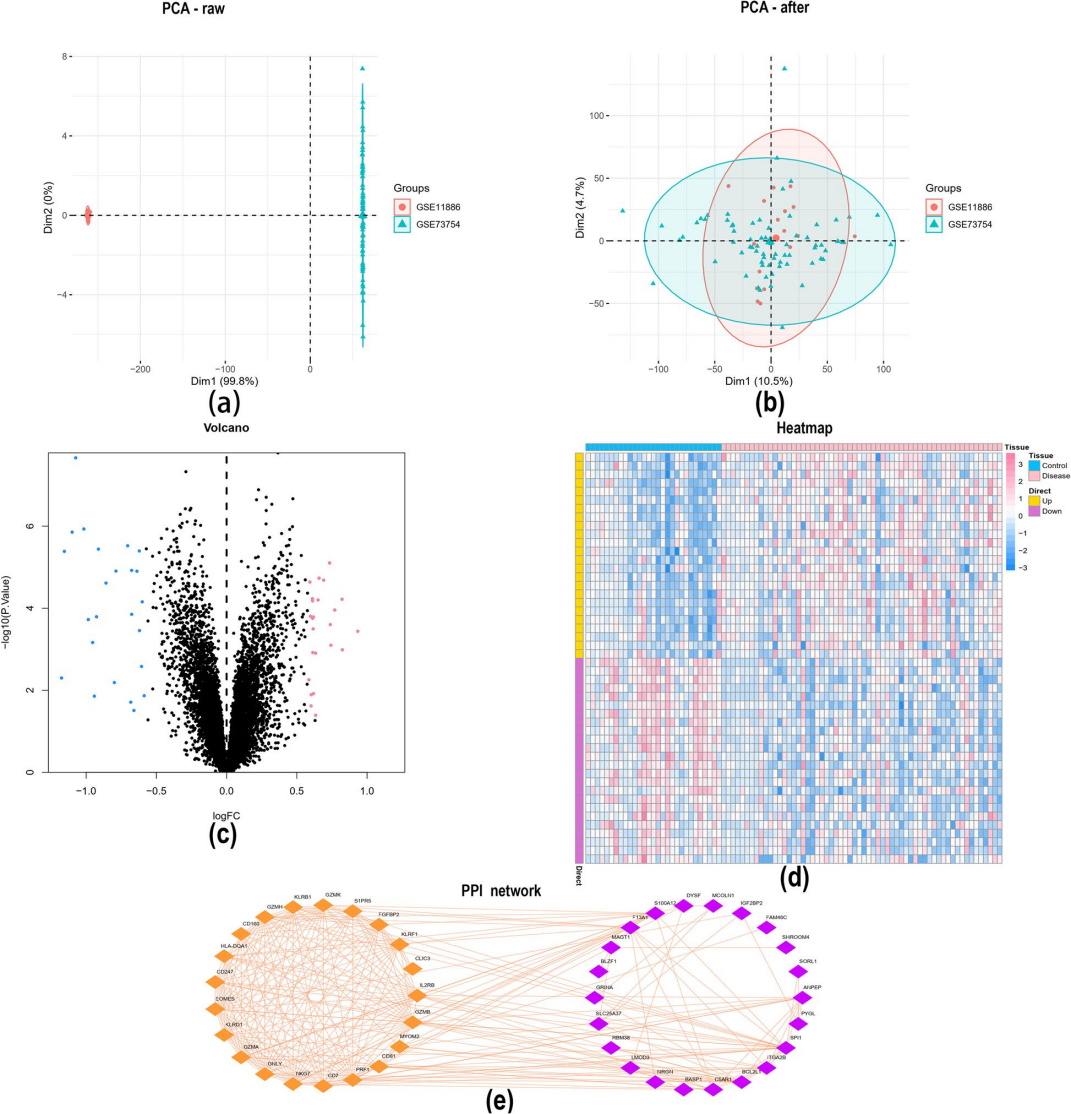
Identification of differentially expressed genes in AS. **a** and **b** The interchip batch effect was reduced after SVA algorithm correction. **c** and **d** Volcano plot and heatmap of DEGs between normal and disease groups, with differentially expressed gene screening conditions of *P* < 0.05 and |logFC|> 0.585; (**e**) PPI network of DEGs

We downloaded the GSE73754 (Table S1) and GSE11886 (Table S2) ankylosing spondylitis-related datasets from the GEO database, which included expression profile data from 89 groups of patients, including the normal group (n = 29) and the disease group (n = 60). The microarray data were corrected using the SVA algorithm. The results corrected by the SVA algorithm showed that the batch effect between chips was reduced (Fig. 1a, b). We next calculated DEGs between the AS-combined control groups using the limma package in R language. According to the *p* < 0.05 and | logFC |> 0.585 standard for differences in gene screening, 48 different genes were finally selected (Fig. 1c-d). Among them, 24 genes were highly expressed, and 24 genes were expressed at low levels. We next searched the PPI network of differentially expressed genes using the String database and visualized the PPI network through Cytoscape. (Fig. 1e).

### Functional enrichment analyses of DEGs

**Fig. 2:**
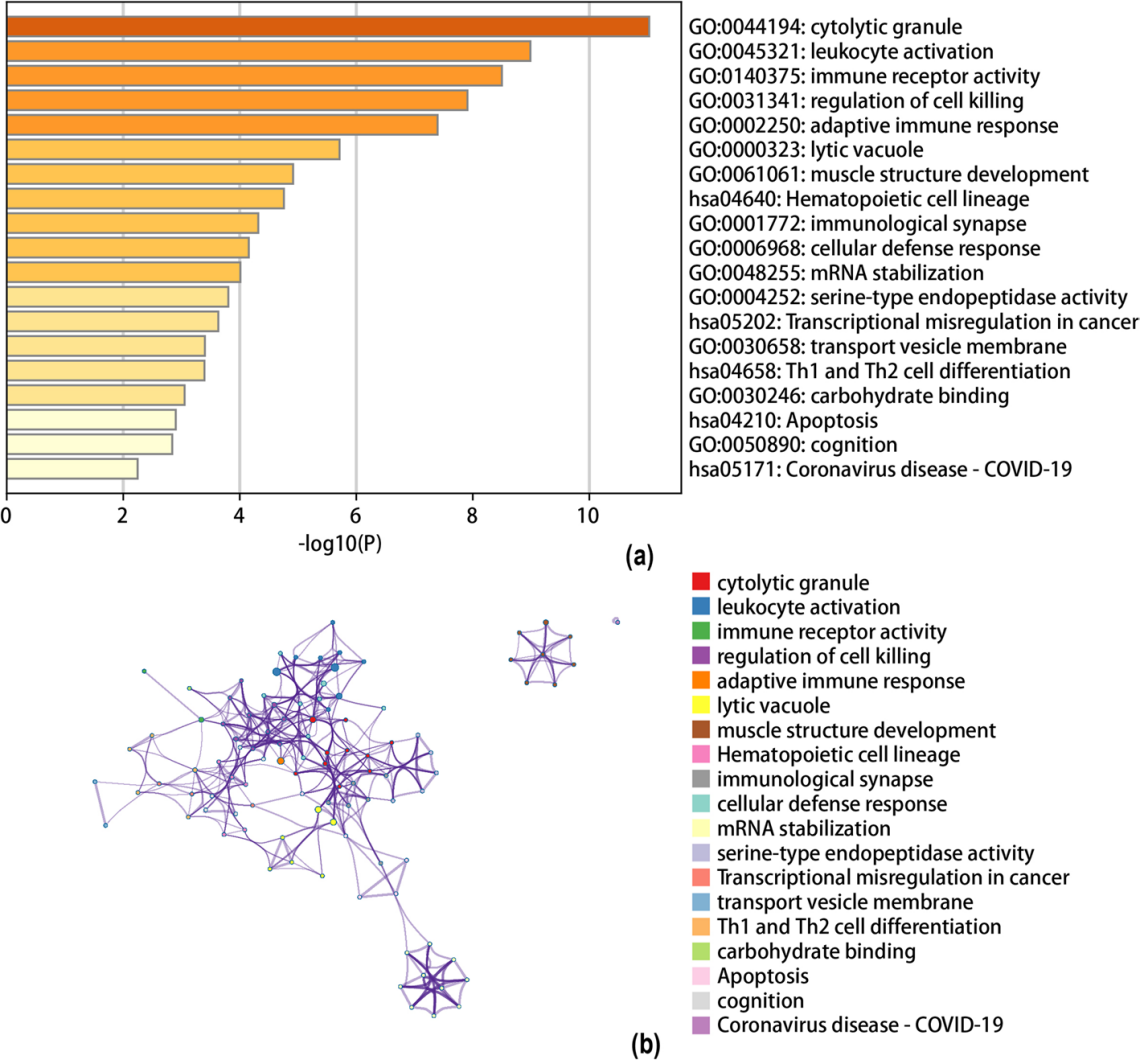
Functional enrichment analysis of DEGs

Next, we performed pathway analysis of DEGs using the Metascape (www.metascape.org) database. Finally, the major GO pathways enriched by DEGs were cytolytic granule, leukocyte activation, immune receptor activity regulation of cell killing, adaptive immune response and other pathways; the enriched KEGG pathways were apoptosis and haematopoietic cell lineage (Fig. 2).

### WGCNA

**Fig. 3:**
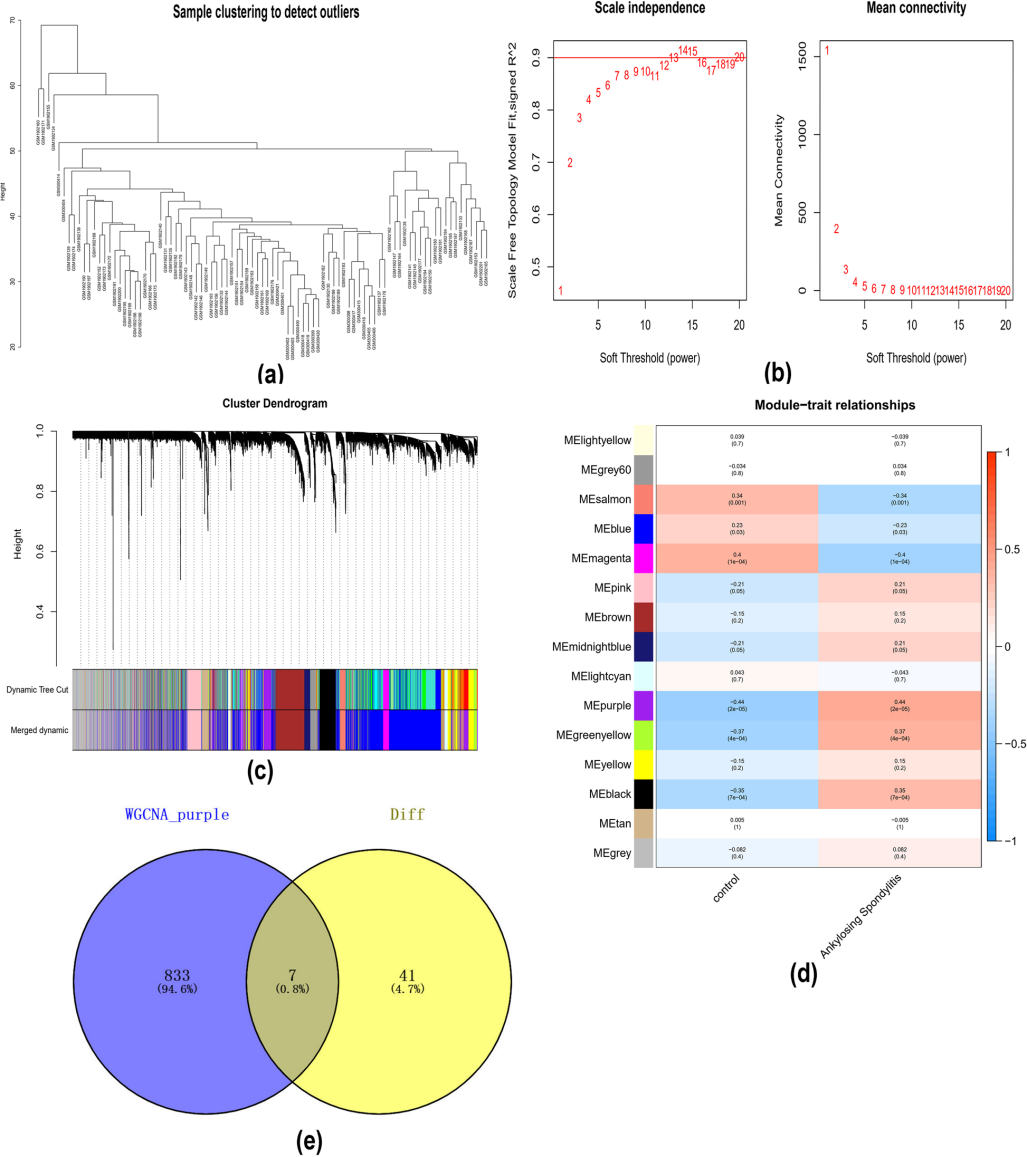
WGCNA. **a** Clustering heatmap of the control and AS groups; (**b**) scale-free exponent and mean connectivity; (**c**) dendrogram of gene clusters; (**d**) heatmap of the correlations. The purple module had the highest correlation. (**e**) A Venn diagram was generated to show the intersection of the purple module with differentially expressed genes

To further identify the core genes affecting ankylosing spondylitis, we constructed WGCNA networks by combining expression profile data to explore relevant coexpression networks in the disease. The soft threshold was set to 7, and then, the gene modules were detected according to the TOM matrix (Table S3). The correlations between modules and traits were further analysed, and we finally found the highest correlation for the purple module (cor = 0.44, *p* = 2e-05). We further intersected the differentially expressed genes with the genes in the purple module and obtained seven intersecting genes: DYSF, BASP1, PYGL, SPI1, C5AR1, ANPEP, and SORL1 (Fig. 3a-e).

### ROC curves for the seven key genes

**Fig. 4:**
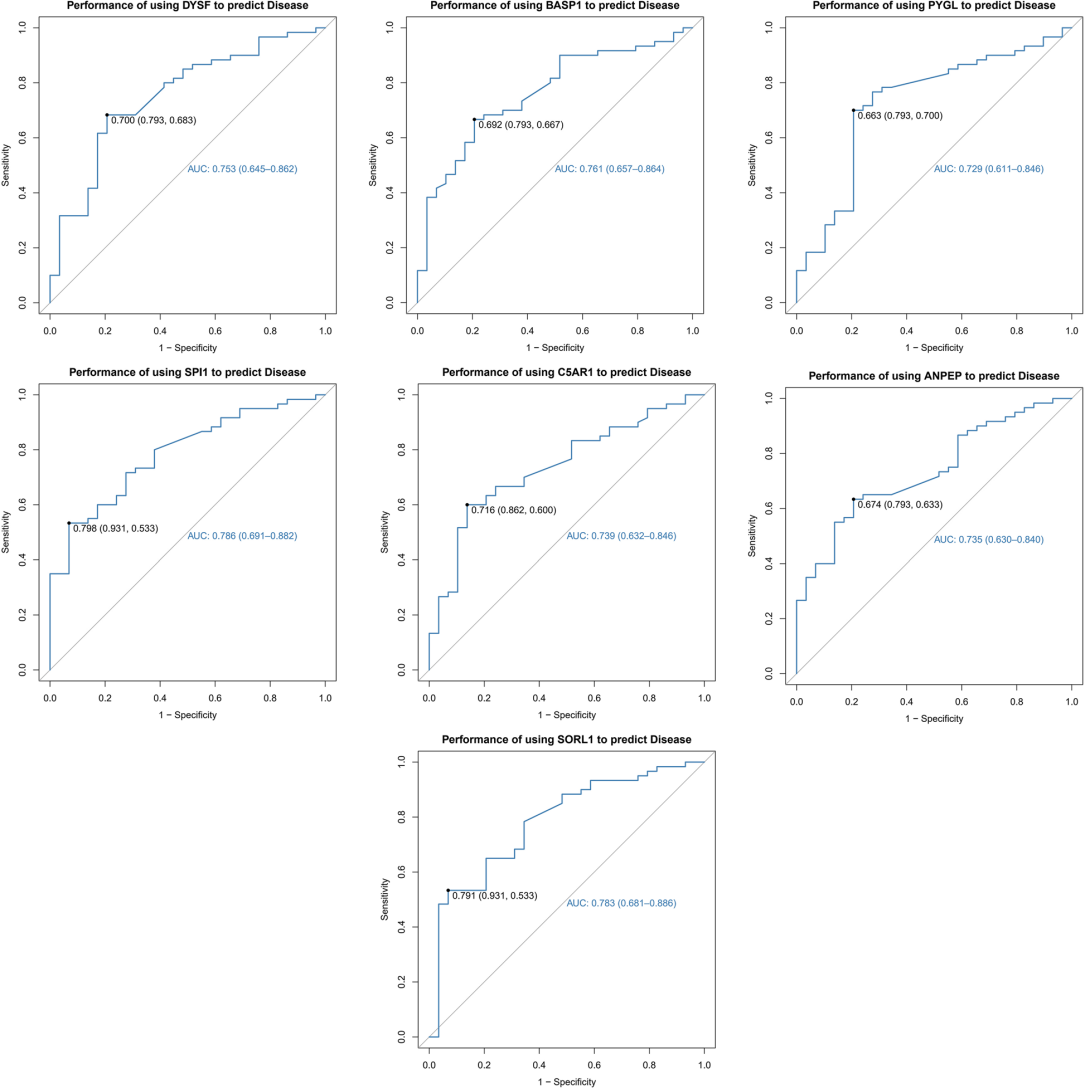
Predictive efficacy of key genes for the disease. The AUC values of the seven key genes suggest their good predictive performance for AS

The diagnostic efficacy was verified by ROC curve analysis. A high AUC represents a good prediction effect. The AUC values for the seven key genes were as follows: DYSF-AUC: 0.753 (0.645–0.862), BASP1-AUC: 0.761 (0.657–0.864), PYGL-AUC: 0.729 (0.611–0.846), SPI1-AUC: 0.786 (0.691–0.882), C5AR1-AUC: 0.739 (0.632–0.846), ANPEP -AUC: 0.735 (0.630–0.840) and SORL1-AUC: 0.786 (0.681–0.886). The AUC values for the seven candidate biomarkers suggest their good predictive performance for AS. (Fig. 4).

### GWAS to confirm the pathogenic regions of core genes in ankylosing spondylitis

**Fig. 5:**
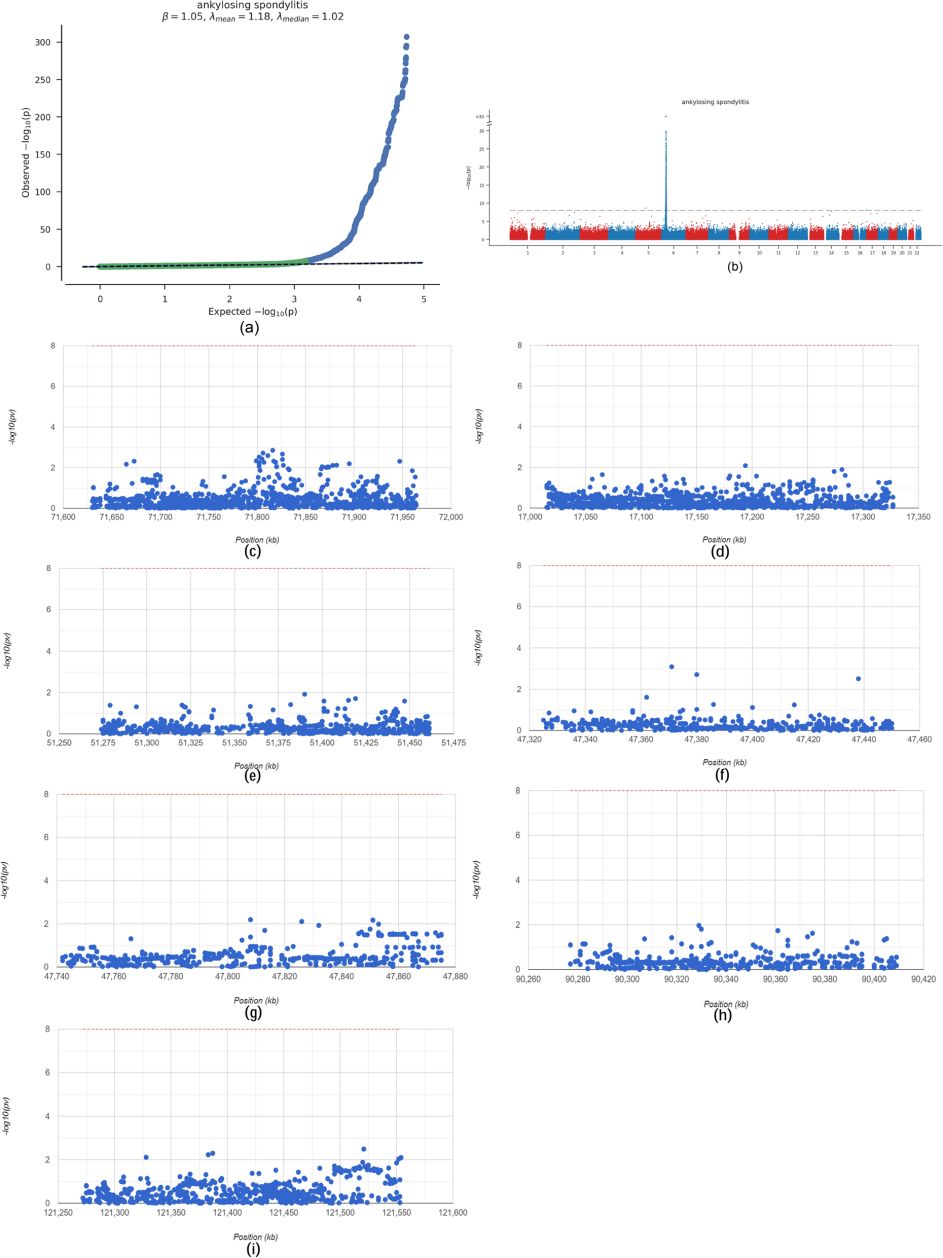
Confirmation of key genes in the pathogenic region of ankylosing spondylitis using GWAS data. **a** Q-Q plot showing significant single nucleotide polymorphism (SNP) loci associated with AS; (**b**) key SNP loci distributed in the enriched region; (**c**-**i**) SNP pathogenic regions corresponding to each of the seven genes (DYSF, BASP1, PYGL, SPI1, C5AR1, ANPEP, SORL1)

Next, we analysed GWAS data for ankylosing spondylitis to identify the pathogenic regions of 7 key genes in ankylosing spondylitis. As shown in Fig. 5A, the Q-Q plots show the significant single nucleotide polymorphism (SNP) loci associated with ankylosing spondylitis identified in the GWAS data (Fig. 5a). The key SNP loci distributed in the enriched region were described by precise spotting of the GWAS data (Fig. 5b). The SNP pathogenic regions corresponding to seven genes are shown, including DYSF in the pathogenic region of chromosome 2, BASP1 in the pathogenic region of chromosome 5, PYGL in the pathogenic region of chromosome 14, SPI1 in the pathogenic region of chromosome 11, C5AR1 in the pathogenic region of chromosome 19, ANPEP in the pathogenic region of chromosome 15, and SORL1 in the pathogenic region of chromosome 11 (Fig. 5c-i). The corresponding significant SNP loci for the seven genes are shown in the supplementary materials (Table S4).

### Immune cell infiltration analysis

**Fig. 6:**
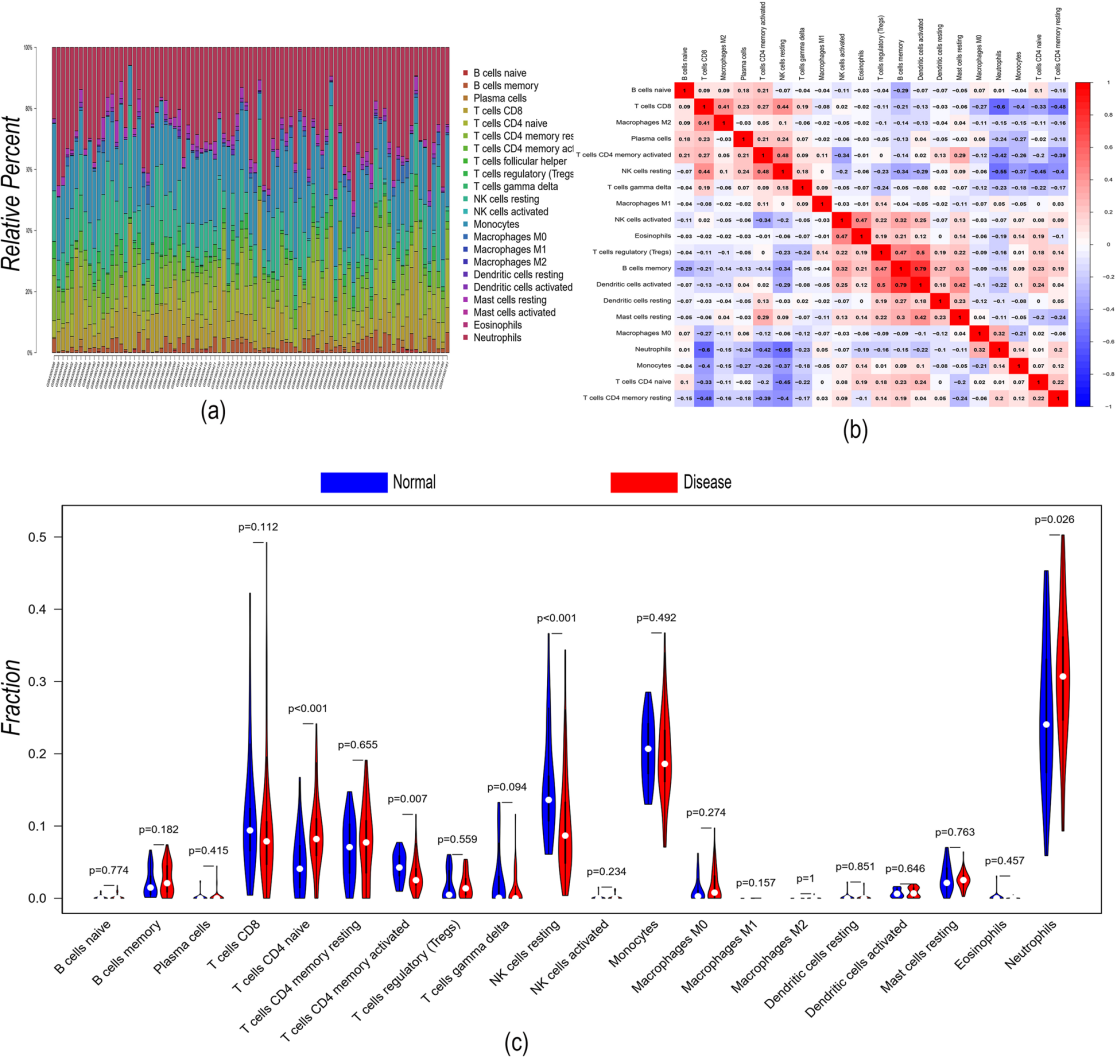
Immune cell infiltration analysis. **a** Immune cell volume; (**b**) Pearson correlation between 22 immune cells. **c** Violin plot of the differences in infiltrating immune cells between the AS (red) and control (blue) groups

**Fig. 7:**
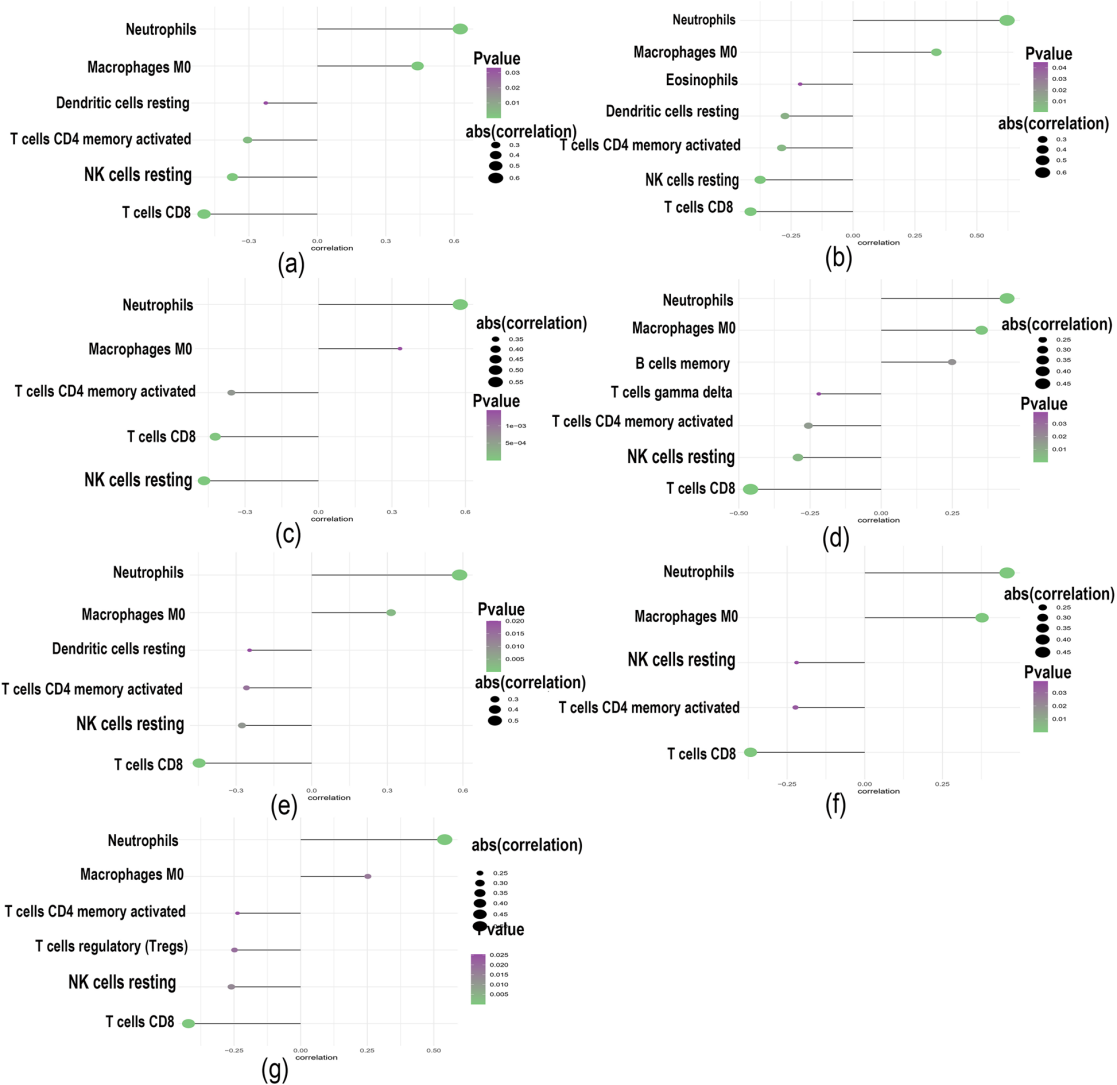
Correlation between key genes (DYSF, BASP1, PYGL, SPI1, C5AR1, ANPEP, SORL1) and immune cells (a–g)

The role of the immune microenvironment in disease diagnosis and prognosis is important in clinical practice. By analysing the relationship between the key genes and immune infiltrating cells in the dataset, the mechanism by which the key genes affect the progression of ankylosing spondylitis was identified. The immune cell content of each patient is shown in Fig. 6a. There were multiple significant correlation pairs between immune infiltration levels (Fig. 6b). The CD4 naive T-cell and neutrophil levels were significantly increased in the AS group (Fig. 6c). We performed Spearman correlation analysis of 7 core genes with immune cells and found that these 7 key genes had a strong correlation with immune cells (Fig. 7a-g). This indicates that the key genes we obtained are closely related to the degree of infiltration of immune cells and play an important role in the immune response.

### Signalling pathways associated with the characteristic genes

**Fig. 8:**
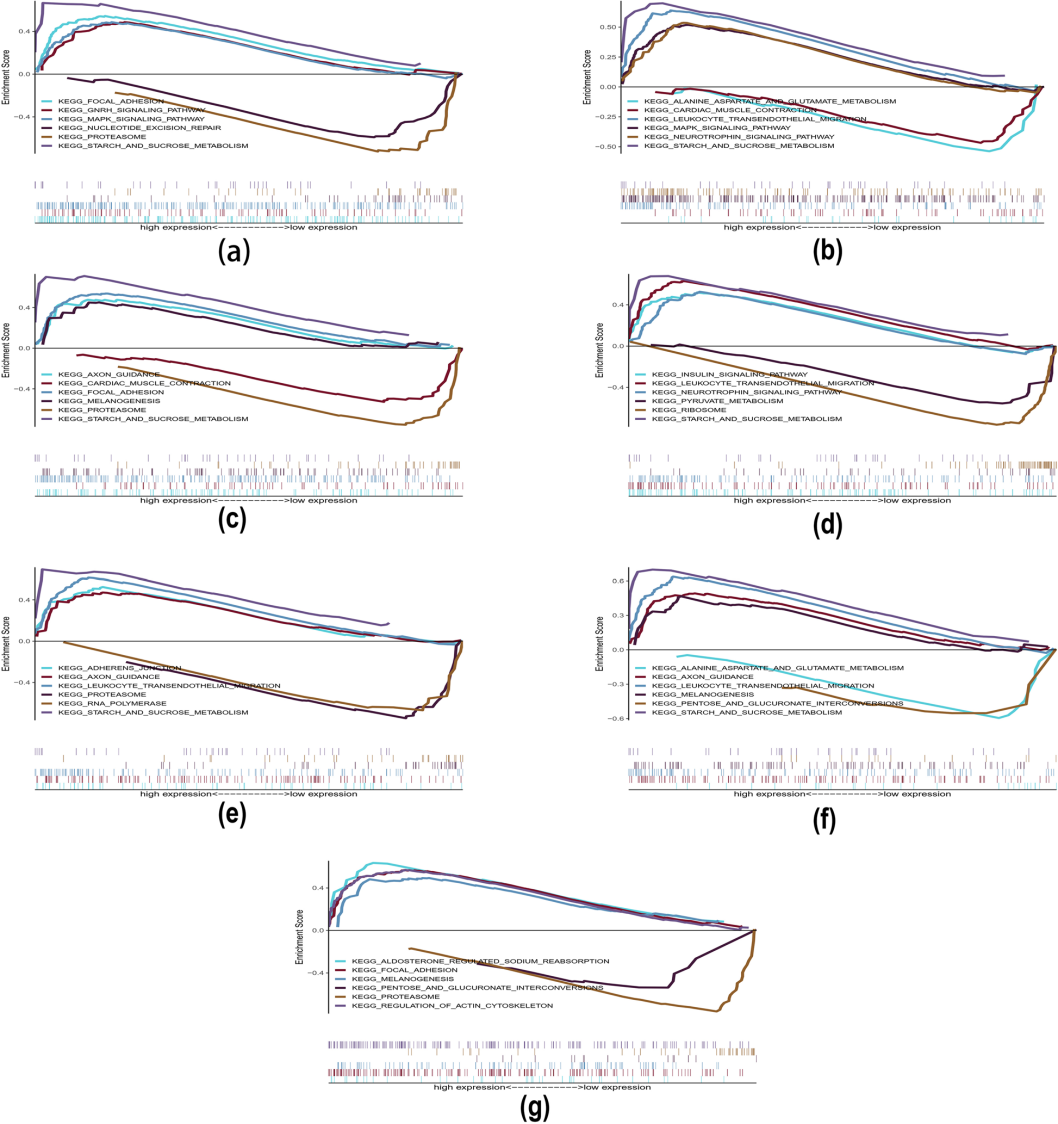
GSEA. **a**-**g** Specific signalling pathways associated with the seven key genes (DYSF, BASP1, PYGL, SPI1, C5AR1, ANPEP, and SORL1)

We further performed GSEA to determine which signalling pathways the key genes were involved in. The results of GSEA showed that the main enriched pathways of DYSF were KEGG FOCAL ADHESION, KEGG GNRH SIGNALING PATHWAY, KEGG MAPK SIGNALING PATHWAY, and KEGG STARCH AND SUCROSE METABOLISM SIGNALING PATHWAY. The main enriched pathways of BASP1 were KEGG LEUKOCYTE TRANSENDOTHELIAL MIGRATION, KEGG MAPK SIGNALING PATHWAY, KEGG NEUROTROPHIN SIGNALING PATHWAY, and KEGG STARCH AND SUCROSE METABOLISM. The main enriched pathways of PYGL were KEGG AXON GUIDANCE, KEGG FOCAL ADHESION, KEGG MELANOGENESIS, and KEGG STARCH AND SUCROSE METABOLISM. The main enriched pathways of SPI1 were KEGG AXON GUIDANCE, KEGG FOCAL ADHESION, KEGG MELANOGENESIS, and KEGG STARCH AND SUCROSE METABOLISM. The main enriched pathways of C5AR1 were KEGG ADHESION, KEGG FOCAL ADHESION, KEGG MELANOGENESIS, and KEGG STARCH AND SUCROSE METABOLISM. The main enriched pathways of ANPEP were KEGG AXON GUIDANCE, KEGG LEUKOCYTE TRANSENDOTHELIAL MIGRATION, KEGG MELANOGENESIS, and KEGG STARCH AND SUCROSE METABOLISM. Finally, the main enriched pathways of SORL1 were KEGG ALDOSTERONE REGULATED SODIUM REABSORPTION, KEGG FOCAL ADHESION, KEGG MELANOGENESIS, and KEGG REGULATION OF ACTIN CYTOSKELETON (Fig. 8a-g and Table S5).

### Analysis of the regulatory network of key genes

**Fig. 9:**
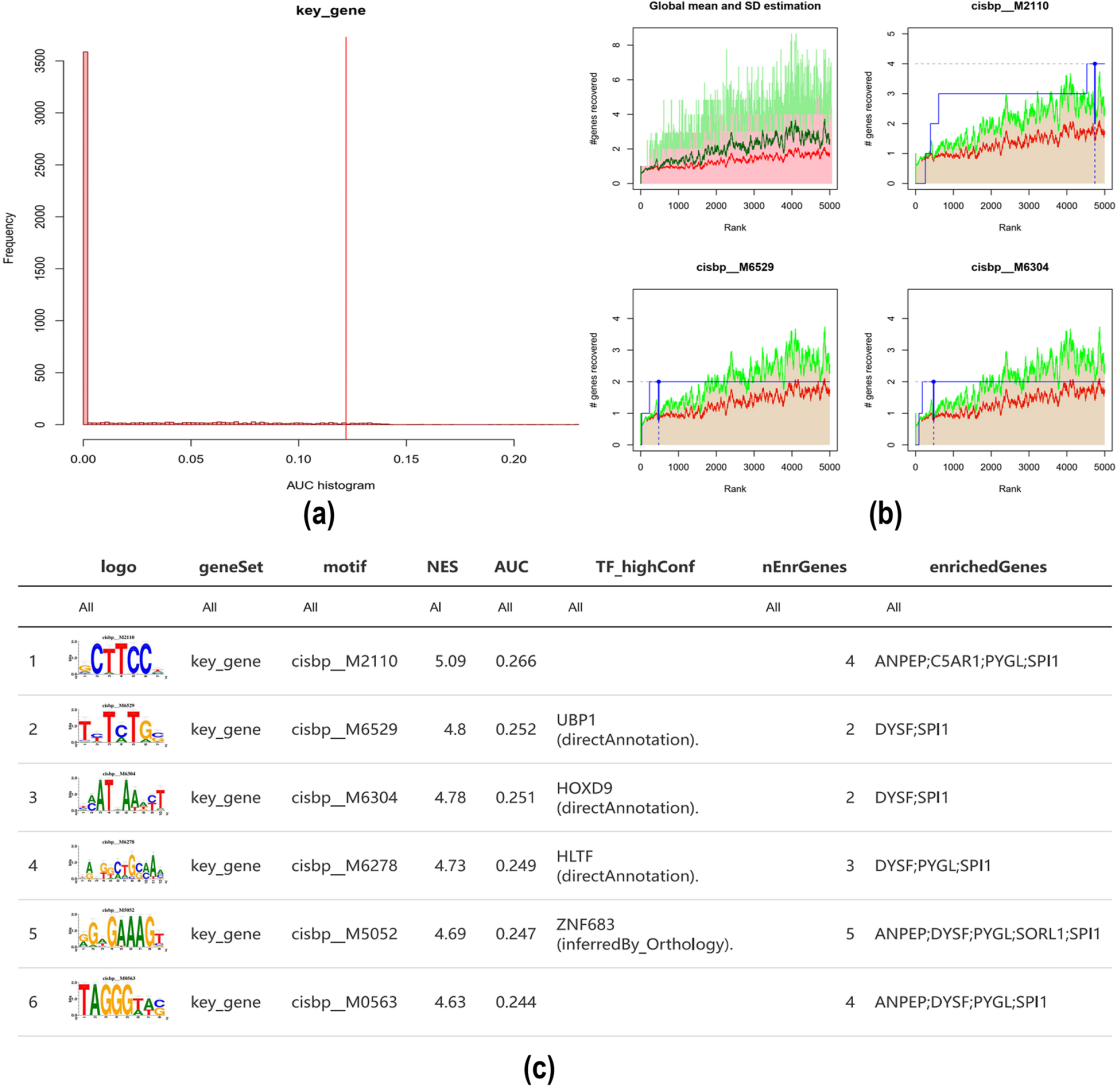
Analysis of key gene regulatory network. **a** and **b** Results of enrichment analysis of cumulative recovery curves. **c** Sequences of enriched genes and their corresponding transcription factors

We analysed the regulatory networks of key genes and showed that they are regulated by multiple transcription factors. Therefore, we used cumulative recovery curves for enrichment analysis of these transcription factors (Fig. 9a-b). Motif-TF annotation and important gene selection analysis showed that cisbp__M2110 was the motif with the highest standardized enrichment score (NES: 5.09). Four key genes were enriched in this motif (Fig. 9c).

### Differential analysis of pathogenic genes in AS

**Fig. 10:**
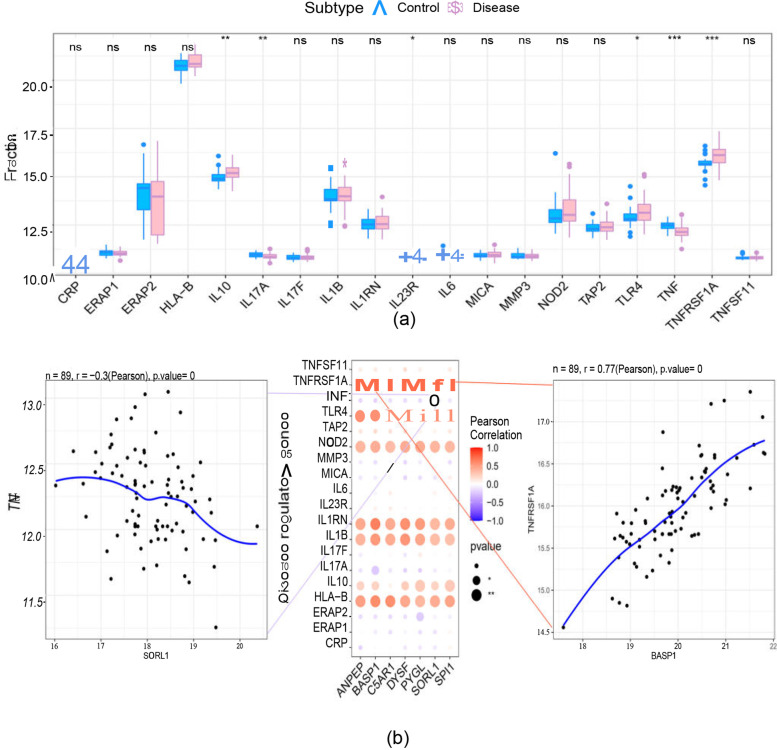
Differential analysis of pathogenic genes in ankylosing spondylitis. **a** Differential expression of AS pathogenic genes between the control (blue) and AS (pink) groups. **b** Correlation analysis between AS-related genes and key genes

**Fig. 11:**
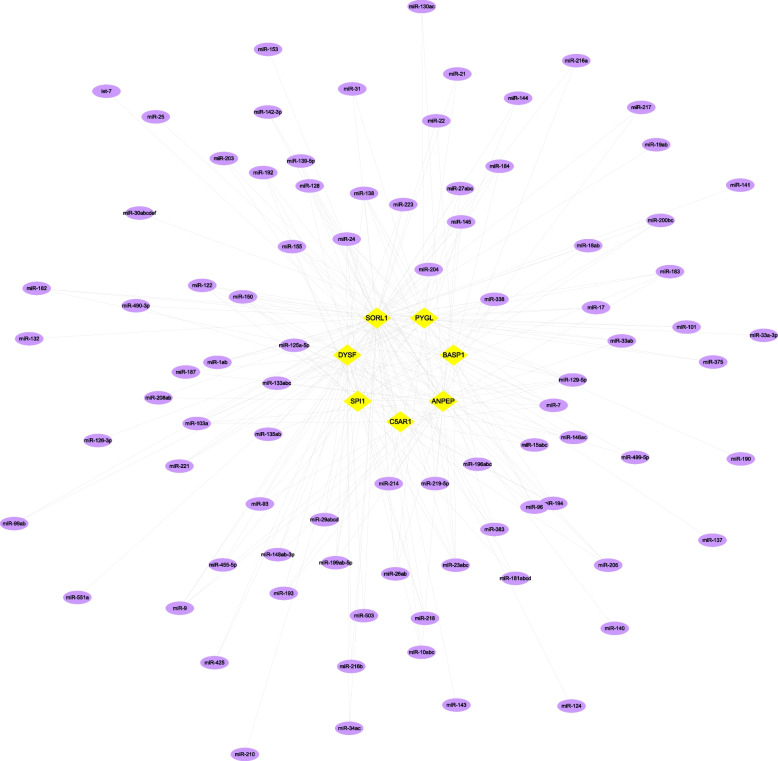
Inverse prediction of seven key genes using the miRcode database yielded 85 miRNAs, with a total of 234 mRNA‒miRNA relationship pairs

We obtained the pathogenic genes associated with ankylosing spondylitis through the GeneCards database. Differential analysis of AS pathogenic genes revealed that the expression of the IL10, IL17A, IL23R, TLR4, TNF, and TNFRSF1A genes differed between the two groups (Fig. 10a). Next, we performed correlation analysis of key genes and regulatory genes of AS, and the expression levels of key genes and several ankylosing spondylitis-related genes were significantly correlated, with SORL1 significantly negatively correlated with TNF (Pearson r = -0.3) and BASP1 significantly positively correlated with TNFRSF1A (Pearson *r* = 0.77). The correlation of disease-related regulatory genes is shown in Fig. 10b. We also reverse predicted 7 key genes using the miRcode database and obtained 85 miRNAs with 234 mRNA‒miRNA relationship pairs (Fig. 11).

### Prediction of potential drugs against AS

**Fig. 12:**
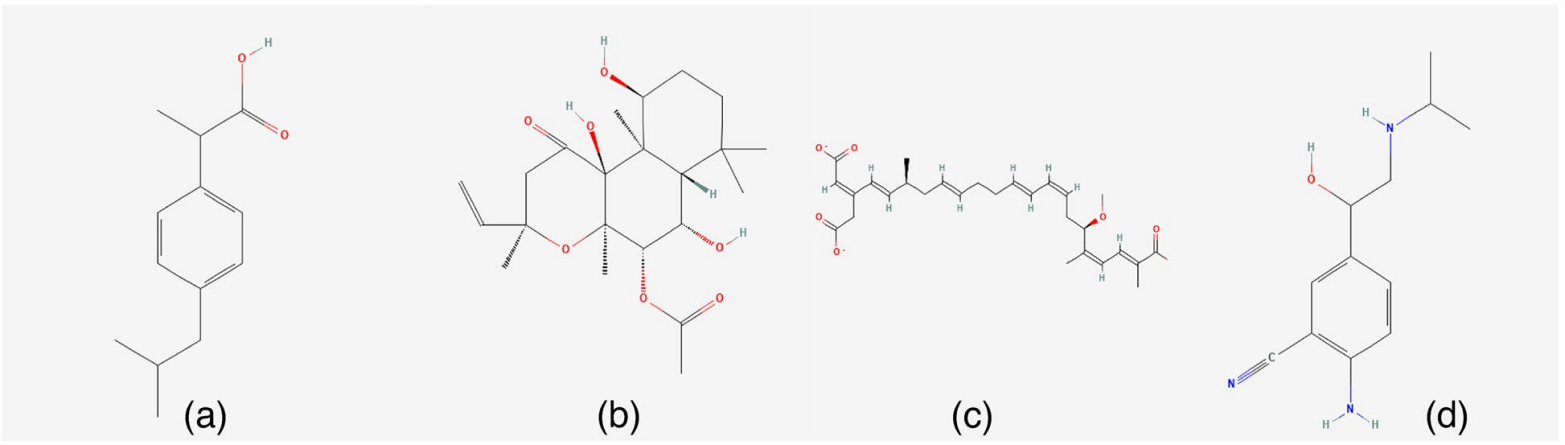
Chemical structures of the four potential drugs: (**a**) ibuprofen; (**b**) forskolin; (**c**) bongkrek-acid; (**d**) cimaterol

In addition, differentially upregulated and downregulated genes were used in the Connectivity Map database for drug prediction, and the results showed that the expression profiles of ibuprofen, forskolin, bongkrek-acid, and cimaterol drug perturbations were most significantly negatively correlated with the expression profiles of disease perturbations, suggesting that the drugs are useful for inhibiting or even reversing the progression of AS (Fig. 12). Drug prediction results are in the supplementary material (Table S6).

## Discussion

AS is a genetic disease with a complex pathogenesis and poor treatment outcome. Although there are many studies on AS in which associated episodic or susceptibility genes have been reported, the pathogenesis is still unclear and needs to be further explored. Among the many susceptibility genes for AS, HLA-B27 is by far the most important [1, 16]. There are two most likely explanations for the pathogenic mechanism of HLA-B27 in AS: one may be its involvement in antigen presentation and arthritogenic peptide production, and the other may be its involvement in endoplasmic reticulum stress and the autophagic response [17]. However, the fact that many individuals with human leukocyte antigen B27 will never develop ankylosing spondylitis suggests that there might be other susceptibility factors that influence the development and progression of AS [18]. Therefore, further searching for other potential susceptibility factors is needed. This study explores the potential molecular mechanisms of AS using a comprehensive bioinformatics analysis approach to provide ideas for new treatment strategies.

Many previous studies have found that certain immune-related pathways are significantly enhanced in AS patients [19, 20]. Consistently, in our study, the results of functional enrichment analysis showed that DEGs were significantly associated with immune-related functions and inflammatory signals. The enriched KEGG pathways were cytolytic granule and leukocyte activation. The enriched KEGG pathways were cytolytic granule, leukocyte activation, immune receptor activity, regulation of cell killing, and adaptive immune response. The enriched KEGG pathways were apoptosis and haematopoietic cell lineage. These results suggest that the immune response plays a key role in the course of AS.

Focus must be placed on the immune microenvironment in the diagnosis and treatment of diseases. To further explore the potential mechanisms of key genes in AS, using the CIBERSORT algorithm, we found that naive CD4 T-cell and neutrophil levels were significantly increased in the disease group samples. Our study also found that most of the key genes were positively correlated with neutrophils and M0 macrophages and negatively correlated with resting NK cells and CD8 T cells, further confirming that immune disorders are important for the progression of AS.

CD4 + T cells have been implicated in the pathogenesis of many types of inflammatory arthritis and produce the pro-inflammatory cytokine IL-17 (Th17) [21]. The reciprocal influence between CD4 + T cells and human leukocyte antigen B27 leads to a cascade of chemokines and cytokines that promote inflammatory responses and bone erosion in AS [22]. Previous studies have confirmed that naive CD4 + /CD8 + T cells are increased, while memory CD4 + /CD8 + T cells and terminally differentiated CD4 + /CD8 + cells are decreased in AS patients [23], and the course of disease has been found to be positively correlated with the initial CD4 + level [24]. TNF-α inhibitors, clinically used anti-AS drugs, act by increasing the proportion of negative regulatory T cells and decreasing the proportion of naive CD4 + T cells in AS patients. (e.g., Tregs and Bregs) [23, 25]. Leukocytes are an important component of the innate immune system and usually play a key role in the chronic inflammatory response of autoimmune diseases [26], and GSEA results indicated that most key genes were enriched in the leukocyte migration pathway, consistent with previously reported results [27, 28].

According to previous reports, glycogen phosphorylase (PYGL) and complement component 5a receptor (C5aR1) promote the inflammatory response in psoriasis [29]. Neutrophils can store large amounts of glycogen, and inhibition of PYGL reduces the number of neutrophil extracellular traps [30]. C5a/C5aR1 is essential for neutrophil re-recruitment in tissues in response to immunoglobulin autoantibody deposition [31]. The results from Zheng et al. showed that C5a/C5aR1 signalling enhanced the recruitment of plasmacytoid dendritic cells, monocytes and neutrophils [32]. Sortilin-related receptor 1 (SORL1) is now known to be closely related to the pathogenesis of familial and sporadic Alzheimer's disease [33, 34]. A recent study found that SORL is closely associated with AS and may be a key factor in the pathogenesis of AS. In that study, neutrophil counts were significantly higher in AS patients than in controls, and neutrophil counts were positively correlated with the expression of SORL. This is consistent with our findings [35]. In the past, desmoplakin (DYSF) has been studied as one of the most common subgroups causing dysferlinopathy (autosomal recessive limb-girdle muscular dystrophy) [36]. STAT3 has been reported to be a possible upstream regulator of DYSF [37], and the two are strongly correlated. STAT3 is involved in the regulation of Th17 cell development and thus in activation of the IL-23/IL-17 axis and contributes to the development of several inflammatory diseases [38, 39]. It has recently been reported that STAT3 and SPI1 may be involved in osteoblast differentiation and bone formation in AS patients through the MAPK signalling pathway, JAK/STAT and Wnt receptors [40]. The GSEA results showed that the DYSF gene was enriched in the MAPK signalling pathway, suggesting that DYSF may be involved in osteoblast differentiation and bone formation in AS. In addition, SPI1 plays an important role in the osteoblast differentiation of dental pulp stem cells by binding to the noggin promoter and repressing noggin expression [41]. This may explain the occurrence of ectopic ossification in AS, which in turn leads to spinal fusion. Recently, many researchers have found that PU.1, encoded by the SPI1 gene, affects the differentiation and function of a variety of myeloid cells and plays an important role in the transcriptional control of certain immune cells and susceptibility to immune diseases [42, 43]. For example, PU.1 can facilitate the progression of rheumatoid arthritis by inducing fibroblast-like synoviocytes and inhibiting macrophages [44] and promotes the expression of pro-inflammatory cytokines by inhibiting miR-150 in autoimmune encephalitis macrophages [45]. Brain acid-soluble protein 1 (BASP1) is highly expressed in brain tissue and promotes brain tissue development by participating in axon regeneration. In essence, Basp1 is a membrane-bound protein [46, 47] that plays a role in apoptosis, differentiation and transcriptional regulation and is a potential tumour suppressor [48, 49]. As an enzyme, ANPEP (CD13) plays an important role in the pathogenesis of multiple inflammatory diseases by regulating the activity of several cytokines through cleavage of the N-terminus and by cutting down the polypeptides bound to MHC II involved in antigen processing, which can regulate the development and activity of immune cells [50].

The GSEA results revealed that key genes may influence the development of AS through multiple biological processes and signalling pathways. These pathways mainly include leukocyte transendothelial migration, focal adhesion signalling pathway, MAPK signalling pathway, axon guidance, and neurotrophic factor signalling pathway. These results suggest that the pathogenesis of AS is closely related to the immune and inflammatory responses, and lay the foundation for further studies of AS immunotherapy.

Through differential analysis of known ankylosing spondylitis morbigenous genes in the Gene Cards database, we found that the expression of the IL10, IL17A, IL23R, TLR4, TNF, and TNFRSF1A genes differed between the two groups of patients, with BASP1 expression levels significantly and positively correlated with TNFRSF1A expression levels (Pearson r = 0.77), and that SORL1 was significantly negatively correlated with TNF (Pearson r = -0.3). A total of 85 miRNAs and 234 mRNA‒miRNA relationship pairs were obtained via reverse prediction of 7 core genes through the miRcode database. However, more studies are required to explore the specific molecular mechanisms involved.

Genetics plays a significant role in the pathogenesis of AS, and in recent years, there has been an increasing focus on genetic factors in AS, leading to the discovery of several drugs [51]. The Gene Atlas genetic mapping database uses the UK Biobank cohort to document relevance between hundreds of traits and millions of variants, with ankylosing spondylitis having the greatest SNP genetic power and a high susceptibility [11]. Disease-associated variants can be identified through GWAS, and GWAS have recorded more than 100 motifs, including those involved in antigen presentation, Th17 response, macrophages and T cells, especially HLA-B27 and ERAP1, with strong associations with AS pathogenesis [52–54]. It is difficult to identify the SNP sites of pathogenic genes, which limits the translation of genetic research results to clinical practice [55]. In this study, we analysed GWAS data in ankylosing spondylitis and identified the pathogenic regions of seven core genes in ankylosing spondylitis, described the key SNP loci distributed in the enriched regions, and demonstrated the SNP pathogenic regions corresponding to the seven genes.

We used the Connectivity Map database to predict potentially useful drugs for AS treatment and found that the expression profiles of ibuprofen, forskolin, bongkrek-acid, and cimaterol drug perturbations were most significantly negatively correlated with the expression profiles of disease perturbations. These results indicate that these drugs may have an inhibitory effect on the progression of AS.

It must be admitted that our research has certain limitation. First, our results were based on bioinformatics analysis of public databases, which may have incomplete or poorly updated data. Second, we have not yet validated our results with clinical samples or cellular assays. It is anticipated that further studies will include additional clinical samples and our results will be validated in laboratory analyses.

## Conclusions

Through the present bioinformatics analysis, we identified seven key genes as potential markers of AS and further explored the various biological functions and pathways through which they affect AS progression, especially playing an important role in immune-related pathways. This provides a new direction for further exploring the pathogenesis of AS and improving the diagnosis and management of AS cases.

## Supplementary Information

**Additional file1: Table S1.** Dataset GSE73754. **Table S2.** Dataset GSE11886. Table S3.Gene profile of each module in WGCNA analysis. **Table S4.** The significance SNP sites corresponding to the sevengenes. **Table S5.** GSEA pathways forkey genes. **Table S6.** Drug predictionresults.

## Data Availability

This study analyses publicly available datasets. These data can be found at GSE73754 and GSE11886 (https://www.ncbi.nlm.nih.gov/geo/).

## Abbreviations

AS: Ankylosing spondylitis
DEGs: Differentially expressed genes
GEO: Gene Expression Omnibus
GSEA: Gene set enrichment analysis
GO: Gene Ontology
KEGG: Kyoto Encyclopedia of Genes and Genomes
WGCNA: Weighted gene coexpression network analysis
PPI: Protein‒protein interaction
GWAS: Genome-wide association studies
ROC: Receiver operating characteristic
CMap: Connectivity Map
