# Supplementary material for: Identification of potential biomarkers for ankylosing spondylitis based on bioinformatics analysis
Source: BMC Musculoskelet Disord. 2023 May 24;24:413. doi: 10.1186/s12891-023-06550-3 (PMC10207833; doi:10.1186/s12891-023-06550-3)
Supplement: Supplementary file 1 — Additional file1: Table S1. Dataset GSE73754. Table S2. Dataset GSE11886. Table S3.Gene profile of each module in WGCNA analysis. Table S4. The significance SNP sites corresponding to the sevengenes. Table S5. GSEA pathways forkey genes. Table S6. Drug predictionresults. [file 12891_2023_6550_MOESM1_ESM.zip › Supplementary Information/legend of Table S1~S6.docx]

Table S1. Dataset GSE73754.

Table S2. Dataset GSE11886.

Table S3. Gene profile of each module in WGCNA analysis.

Table S4. The significance SNP sites corresponding to the seven genes. Table S5. GSEA pathways for key genes.

Table S6. Drug prediction results.
